# Antenatal exposure to betamethasone induces placental 11β-hydroxysteroid dehydrogenase type 2 expression and the adult metabolic disorders in mice

**DOI:** 10.1371/journal.pone.0203802

**Published:** 2018-09-13

**Authors:** Li Ni, Yibin Pan, Chao Tang, Wenyi Xiong, Ximei Wu, Chaochun Zou

**Affiliations:** 1 Department of Endocrinology, the Children Hospital, School of Medicine, Zhejiang University, Hangzhou, China; 2 Department of Pharmacology, School of Medicine, Zhejiang University, Hangzhou, China; 3 Jiaxing Maternity and Child Health Care Hospital, Jiaxing, China; University of Southampton, UNITED KINGDOM

## Abstract

Antenatal overexposure to glucocorticoids causes fetal intrauterine growth restriction (IUGR) and adult metabolic disorders. 11β-hydroxysteroid dehydrogenase (11β-HSD) 1 and 2 are key enzymes for glucocorticoid metabolism, however, the detailed effects of antenatal overexposure to glucocorticoids on placental 11β-HSD1 and 2 expression and adult metabolic disorders remain obscure. Here, we report that, in placenta 11β-HSD1 is diffusely localized, whereas 11β-HSD2 is specifically expressed in labyrinthine layer. Exposure of pregnant dams to betamethasone significantly increases the expression of placental 11β-HSD2 but not 11β-HSD1, and decreases the weights of fetuses but not placentas. Antenatal exposure to betamethasone leads to either significant weight loss in the offspring younger than 10-week-old, or weight gain in those older than 14-week-old. Furthermore, antenatal exposure to betamethasone results in coexistence of various metabolic disorders in adult offspring, including hyperglycemia, glucose intolerance, low insulin secretory capacity and hyperlipidemia. The present study demonstrates that exposure of pregnant dams to betamethasone induces the expression of placental 11β-HSD2 but not 11β-HSD1, leads to fetal IUGR and causes adult metabolic disorders, providing evidence for fetal origins of adult diseases and the potential role of placental 11β-HSD2 in them.

## Introduction

11β-hydroxysteroid dehydrogenase (11β-HSD) is a key enzyme for metabolism of glucocorticoids (GC), and two types of 11β-HSD, including11β-HSD1 and 11β-HSD2, have been characterized and successfully cloned from both human and rodent tissues [1, 2]. 11β-HSD1 is expressed in numerous human tissues, such as liver, testis, ovary, lung, and placenta as well, and it converts biologically inert 11-ketoglucocorticoids (cortisone, 11-dehydrocorticosterone) into active 11β-hydroxyglucocorticoids (cortisol, corticosterone). In human placenta, 11β-HSD1 is abundantly localized in decidua where it guarantees enough active GC to maintain local immunosuppressive effects for the blastocyst implantation and immunological tolerance to the alien tissue [1,3,4]. 11β-HSD2 is expressed in mineralocorticoid target tissues, such as kidney, salivary glands, ileum, distal colon, and placenta [5], and it functions as an exclusive dehydrogenase for endogenous steroids (cortisol and corticosterone). In humans, 11β-HSD2 catalyzes their unidirectional conversion to inactive 11-oxo metabolites, converting cortisol to the inactive GC, cortisone; in rodents, it catalyzes corticosterone into dehydrocorticosterone. In human placenta, 11β-HSD2 is selectively expressed in the syncytiotrophoblasts [6], forming a GC barrier to block more than 5~10 folds of maternal GC into fetal blood circulation [7, 8].

Metabolic syndrome is characterized by the variable coexistence of obesity, hyperglycemia, insulin resistance, dyslipidemia and hypertension [9]. A large number of epidemiological studies indicate that adverse pregnant environments lead to intrauterine growth restriction (IUGR), low birth weight as well as premature birth, and these risk factors permanently decide and "code" (programming) metabolic syndrome in adulthood, increasing the incidence of fetal origin of adult diseases (FOAD) including type II diabetes, hyperlipidemia, hypertension and coronary heart disease [9,10]. Among these adverse pregnant environments, exposure of the fetus to excessive GC due to chronic maternal stress plays very important roles in origin of IUGR and low birth weight, and is one of the main factors "imprinting" FOAD [10–12] As GC is important for the development of not only intrauterine but also postnatal fetuses, maternal GC levels in circulation increase and reach values in the range seen in Cushing’s syndrome in normal pregnancy [12,13]. GC levels in intrauterine fetuses are controlled by placental glucocorticoid barrier during gestation. Normally, 11β-HSD2 in the placental syncytiotrophoblast layer catalyzes maternal active cortisol into inactive cortisone, blocking the entering of cortisol to the fetus [14]. However, when the endogenous maternal GC levels are markedly increased under stress, either the high levels of maternal GC exceeding the capacity of placental 11β-HSD2 for inactivation of excessive GC or pathological conditions impairing placental functions could lead to fetal overexposure to GC [15].

It is well established that intrauterine overexposure to GC induces fetal IUGR, hyperglycemia and hypertension in adult offspring [9]. Though placental 11-βHSD expressions are regulated by synthetic GC treatment and GC programming of glucose intolerance in the adult offspring[16–19], the detailed effects of GC overexposure during pregnancy on placental 11β-HSD expression and lipid homeostasis in adult offspring remain obscure. In the present study, we examined the expression patterns for both 11β-HSD1 and 11β-HSD2 in murine placenta in different stages, and then modeled murine IUGR through antenatal exposure to synthetic steroid derivative, betamethasone (BTM), which is not metabolized by 11β-HSD2, and thus can be poured into the fetuses like dexamethasone [2,20–22], to investigate the effects of exposure to BTM on placental 11β-HSD1 and 2 expression and on adult metabolic disorders.

## Materials and methods

### Chemicals and reagents

Betamethasone (CASNo. 378-44-9) was obtained from Melone Pharmaceutical Co., Ltd. (Dalian, China), and streptavidin-horseradish peroxidase (HRP) kit was from CoWin Bioscience Co., Ltd. (Beijing, China). Rabbit polyclonal anti-11β-HSD2 (H-145), anti-β-actin, and anti-α-tubulin antibodies were purchased from Beyotime Institute of Biotechnology, Inc. (Shanghai, China), and rabbit polyclonal anti-11β-HSD1 was purchased from Abcam Ltd. (Cambridge, MA). Rat/mouse insulin ELISA kit was obtained from EMD Millpore Co. (Billerica, MA), and mouse corticosterone ELISA kit was from Abnova Co. (Taipei City, Taiwan). Glucose (GLU) test kit, triglyceride (TG) test kit, total cholesterol (TC) test kit, serum high-density lipoprotein cholesterol (HDL-C) test kit and serum low-density lipoprotein cholesterol (LDL-C) test kit were obtained from Nanjing Jiancheng Bioengineering Institute (Nanjing, China). All other chemicals were of reagent grade.

### Animals

Eight-week-old ICR mice with the weights of 22 ± 0.5 g for females and 26 ± 0.6 g for males were purchased from Shanghai Slac Laboratory Animal Co., Ltd. (China, Certificate No. 2007–0005). All mice were housed in a room maintained at 23 ± 2°C with 50 ± 10% humidity and a 12-h light 12-h dark cycle (lights on from 8:00 a.m. to 8:00 p.m.). Mice were allowed free access to tap water and regular rodent chow. All the animal care and handling procedures were approved by the Institutional Animal Care and Use Committee of Zhejiang University ([2016]040). Every two female mice were mated with one male mouse, and the plugged mice were used for further experiments. For timed mating, day 0.5 of pregnancy (E0.5) was defined as the morning on the day a vaginal plug was found after overnight mating.

### Procedure of experiments

To determine the localization and expression of 11β-HSD1 and 2 in murine placentas of different embryonic stages, the placentas were harvested at E11.5, E13.5, E15.5 and E17.5 (or E18.5), respectively. Each time-point contains three pregnant dams. To investigate the effects of exposure to BTM on the expression of 11β-HSD1 and 2 in placentas, we subcutaneously injected the pregnant dams at E8.5, E9.5, E10.5 and E14.5 (or E15.5) with either normal saline (NS) or BTM at 600 μg/kg/d for 4 d. Each group contains twelve pregnant dams (three each time-point). Eight hours after last injection, the placentas were harvested at E11.5, E12.5, E13.5 and E17.5 (or E18.5), respectively, and were subject to preparation of protein samples for determination of 11β-HSD1 and 11β-HSD2 protein levels by western blots. To investigate the effects of exposure to BTM on the fetal and placental weights and metabolic disorders, we subcutaneously injected the pregnant dams with NS or BTM at either 400 or 600 μg/kg/d from E14.5 for 4 d. Each group contains twelve pregnant dams. BTM was dissolved in ethanol. Briefly, 50 mg BTM powder was dissolved with 7.5 ml ethanol to make the BTM solution. When injection, 500 μl BTM solution was diluted by 500 ml saline to make the working solution with the final concentration of 10 mg/150 ml. Eighteen pregnant dams were sacrificed at E18.5 for weighing the fetuses and placentas, and the rest of them (eighteen pregnant dams) underwent further bearing. After weaned, the pups from either NS- or BMT-treated pregnant dams were fed with high-fat diets containing 16.20% calories as fat (M04-F, Shanghai Slac Laboratory Animal Co., Ltd., Shanghai, China), and were weighed and subjected to various assays including glucose tolerance test, serum glucose determination, measurements of insulin secretory capacity, serum corticosterone and lipid determination in different postnatal stages. Mice were euthanized with CO_2_, all efforts were made to minimize suffering. All experiments were repeated three times.

### Immunohistochemistry

After the murine placentas were fixed with 10% neutralized formalin for 24 h, the paraffin-embedded placental sections (6 μm) were prepared, and underwent deparaffinization and rehydration. After that, sections were boiled in Tris-EDTA buffer (pH 9.0) for antigen retrieval, and endogenous peroxidase activity was inactivated by incubation with 0.3% H_2_O_2_ for 20 min. After blocked with rabbit normal serum at room temperature for 60 min, sections were incubated with normal rabbit IgG (negative control), primary rabbit polyclonal anti-11β-HSD2 antibody (1:50) or anti-11β-HSD1 antibody (1:100) overnight at 4°C. After rinsed, sections were incubated with the secondary HRP-labeled goat anti-rabbit IgG for 30 min at room temperature. Then, the sections were subjected to 3, 3'-diaminobenzidine (DAB) chromogenic reaction by incubation for 10 min at room temperature. After termination of the reaction, the sections were counter-stained with hematoxylin and examined using Olympus microscopy.

### Western blot

The protein extraction and western blot were described previously [23, 24]. Briefly, after thawed in lysis buffer (50 mM Tris–Cl, pH7.4, 150 mM NaCl, 1% Triton-X-100, and 0.5 mM EDTA) containing protease inhibitor cocktail (Santa Cruz Biotechnology, Santa Cruz, CA), the placentas were homogenized. The lysates were centrifuged at 13000 g for 30 min at 4°C, and the protein samples (25 μg protein/well) were subjected to SDS-PAGE electrophoresis in a 10% gel, and were transferred to a PVDF membrane. Immunoblotting was performed with rabbit polyclonal 11β-HSD1 and anti-11β-HSD2 antibodies, respectively. A peroxidase-conjugated anti-rabbit IgG was used as a secondary antibody, and the immunoreactive bands were visualized with an ECL system (Thermo Fisher Scientific). Either α-tubulin or β-actin was used as internal controls for quantification of 11β-HSD2 and 11β-HSD1 expression, respectively. Image software from National Institutes of Health was used to quantify the immunoreactive bands (ImageJ sofware, http://rsb.info.nih.gov/ij/download.html), and the normalized antigen signals were calculated from 11β-HSD2-, 11β-HSD1-, α-tubulin-derived or β-actin-derived signals. The mean levels that arose from E11.5 were defined as 1.

### Glucose and insulin determination and glucose tolerance test

After adult offspring at different ages were fasted for 12 h, they were subjected to isolation of sera for determination of glucose levels by a glucose (GLU) test kit according to the manufacture’s instruction. The glucose tolerance test was performed in adult offspring at 16-week-old, after they were fasted for 12 h. Blood was collected from the caudal vein. The sera was isolated for determination of glucose levels right after orally administrated with glucose at 2.0 g/kg for 0, 1, 2 and 4 h, respectively, and the sera was isolated for determination of insulin levels, right after orally administrated with the same dosage of glucose for 0, 15, 30 and 60 min, respectively. The inter-assay and intra-assay coefficients of variation were 5% and 5% for Glucose test kit, and were 3.6% and 4.5% for insulin ELISA kit, respectively.

### ELISA assay for serum corticosterone levels

After fasted for 12 h, the adult offspring at different ages were subjected to isolation of sera for determination of corticosterone levels by ELISA kits according to the manufacture’s instruction. For the corticosterone ELISA kit, the inter-assay and intra-assay coefficients of variation were 3.7% and 4.5%, respectively.

### Determination of serum lipid levels

Serum triglyceride (TG), total cholesterol (TC), high-density lipoprotein cholesterol (HDL-C) and low-density lipoprotein cholesterol (LDL-C) were determined by using TG enzymatic test kit, TC enzymatic test kit, HDL-C test kit and LDL-C test kit, respectively. The inter-assay and intra-assay coefficients of variation were 3% and 5% for TG determination, 3% and 5% for TC determination, 5% and 5% for HDL-C determination, 10% and 10% for LDL-C determination, respectively.

### Statistics

Numerical data were presented as means ± SEM, and were analyzed by two-Way ANOVA and Tukey’s multiple comparisons test with SPSS software (Chicago, IL). Significance was assessed at *p*<0.05 and *p*<0.01 levels. All the experiments were triplicated, and the results were qualitatively identical. The representative results are shown.

## Results

### Expression of 11β-HSD1 and 2 in murine placentas

**Fig 1:**
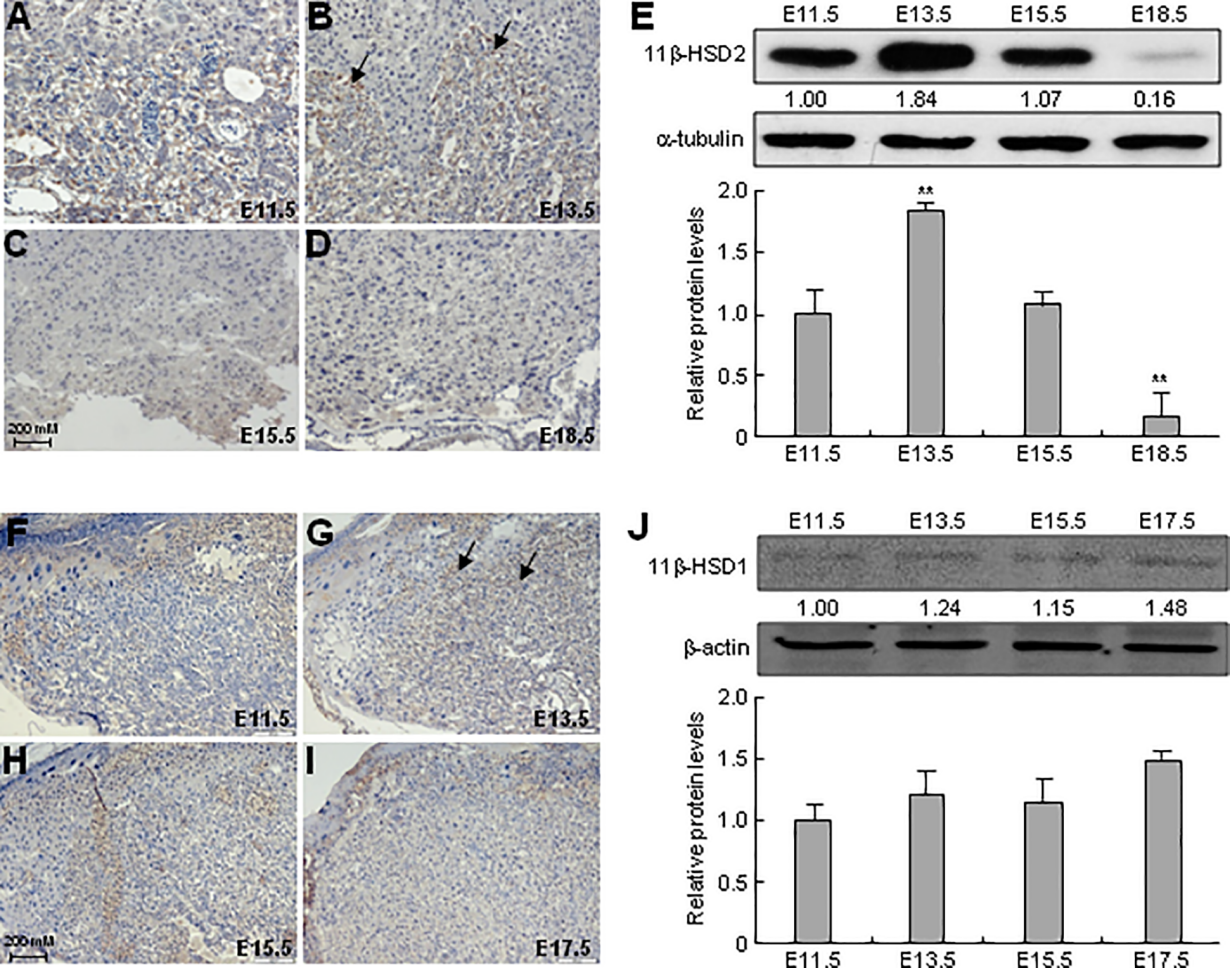
Expression of 11β-HSD1 and 2 in murine placentas in different stages. The murine placentas at E11.5 (**A, F**), E13.5 (**B, G**), E15.5 (**C, H**), E17.5 (**I**) and E18.5 (**D**) were harvested for determination of either 11β-HSD1 or 11β-HSD2 expression by immunohistochemistry stainings (**A-D, F-I**) and western blot assays (**E, J**), respectively. The relative expression of 11β-HSD1 and 2 were normalized by internal control β-actin and α-tubulin values, respectively, the mean levels arose from E11.5 were defined as 1, and quantitative information was provided. Representative data from three independent experiments are shown. Data are expressed as means ± SEM, **p < 0.01 versus E11.5.

To determine the localization and expression of 11β-HSD1 and 2 in murine placentas of different embryonic stages, we performed immunohistochemistry and western blot assays. 11β-HSD2 was specifically localized in the labyrinthine layer of murine placentas, and was expressed at from E11.5 to E18.5 (Fig 1A–1D). And, at E13.5, the expression level of 11β-HSD2 reached a peak (Fig 1A–1D). Consistent with the immunohistochemistry findings, western blot assays revealed that 11β-HSD2 level was increased from E11.5 to E13.5, gradually decreased from E15.5 to E18.5, and reached to almost undetectable one at E18.5 (Fig 1E). In contrast, 11β-HSD1 was mainly distributed in the decidua and spongiotrophoblast layer of murine placenta at E11.5 and E13.5, and was diffusely expressed in both spongiotrophoblast and labyrinthine layers at E15.5 and E17.5 (Fig 1F–1I). Unlike 11β-HSD2, the 11β-HSD1 was expressed almost evenly in different embryonic stages (Fig 1J).

### Effects of BTM on the expression of 11β-HSD1 and 2

**Fig 2:**
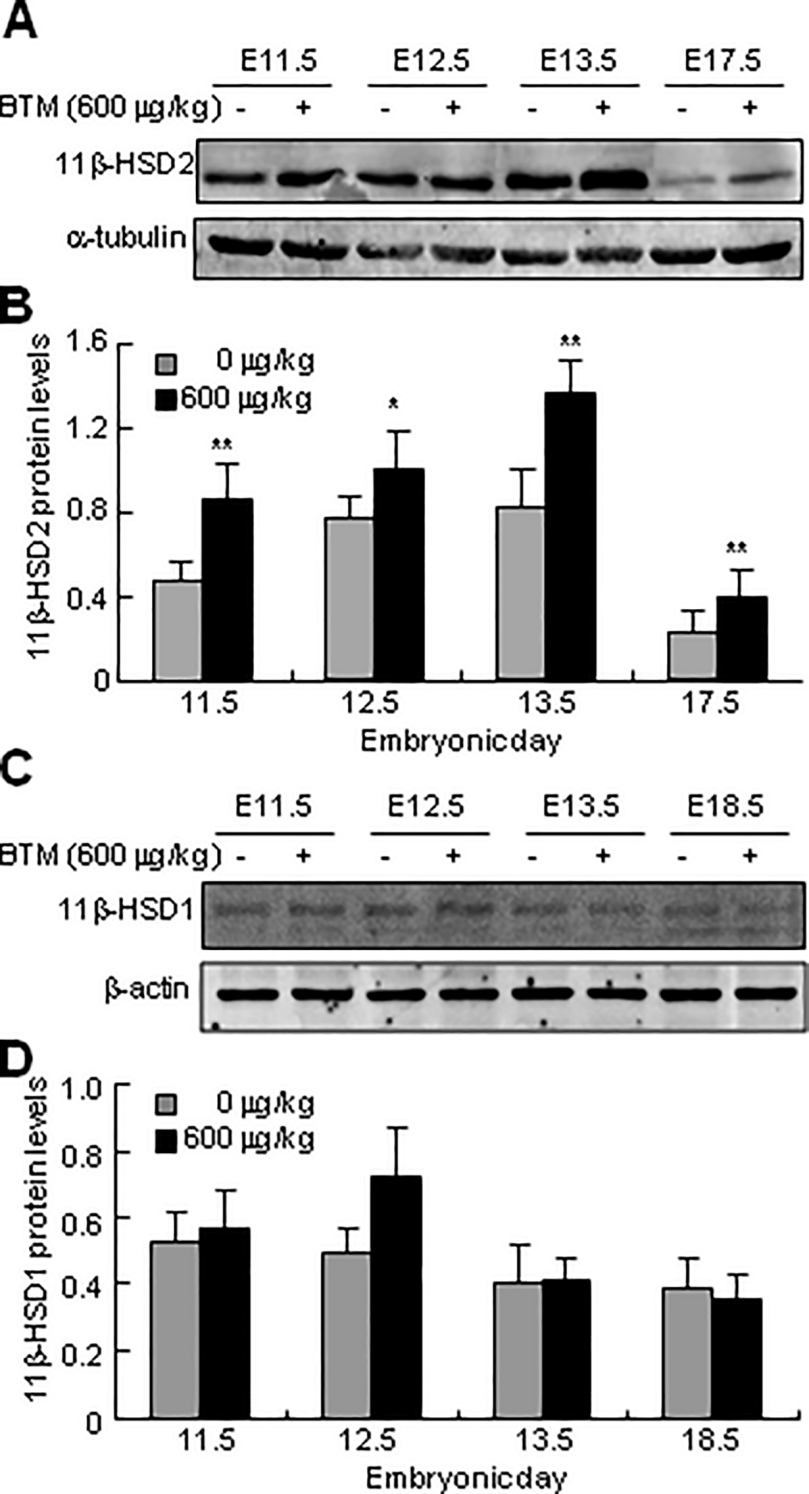
Effects of exposure to BTM on expression of placental 11β-HSD1 and 2. Pregnant dams were subcutaneously injected with normal saline (-) or BTM at 600 μg/kg/d (+) for 4 d, and the placentas were harvested at E11.5, E12.5, E13.5 and either E17.5 or E18.5, respectively. The placentas were further subjected to protein extraction and western blots for determination of 11β-HSD1 and 2 protein levels. The relative expression of 11β-HSD1 and 2 were normalized by internal control β-actin and α-tubulin values, respectively. Data are expressed as means ± SEM (each n = 6 from 3 littermates). *p < 0.05, **p < 0.01 versus exposure to normal saline (-).

Both 11β-HSD1 and 2 are rate-limiting enzymes for GC metabolism. To investigate whether GC affected their expression, we administrated the pregnant dams with either NS or BTM at 600 μg/kg/d for 4 d, and harvested the placentas at E11.5, E12.5, E13.5, and E17.5 (or E18.5) for determination of 11β-HSD1 and 2 protein levels by western blots. Exposure to BTM at 600 μg/kg/d induced placental 11β-HSD2 levels to a different extent from E11.5 to E17.5, and the maximal induction was achieved at E13.5 (Fig 2A and 2B). However, exposure to BTM at 600 μg/kg/d for 4 d did not affect the 11β-HSD1 protein levels at all (Fig 2C and 2D).

### Effects of exposure to BTM on the weights of placentas and offspring

**Fig 3:**
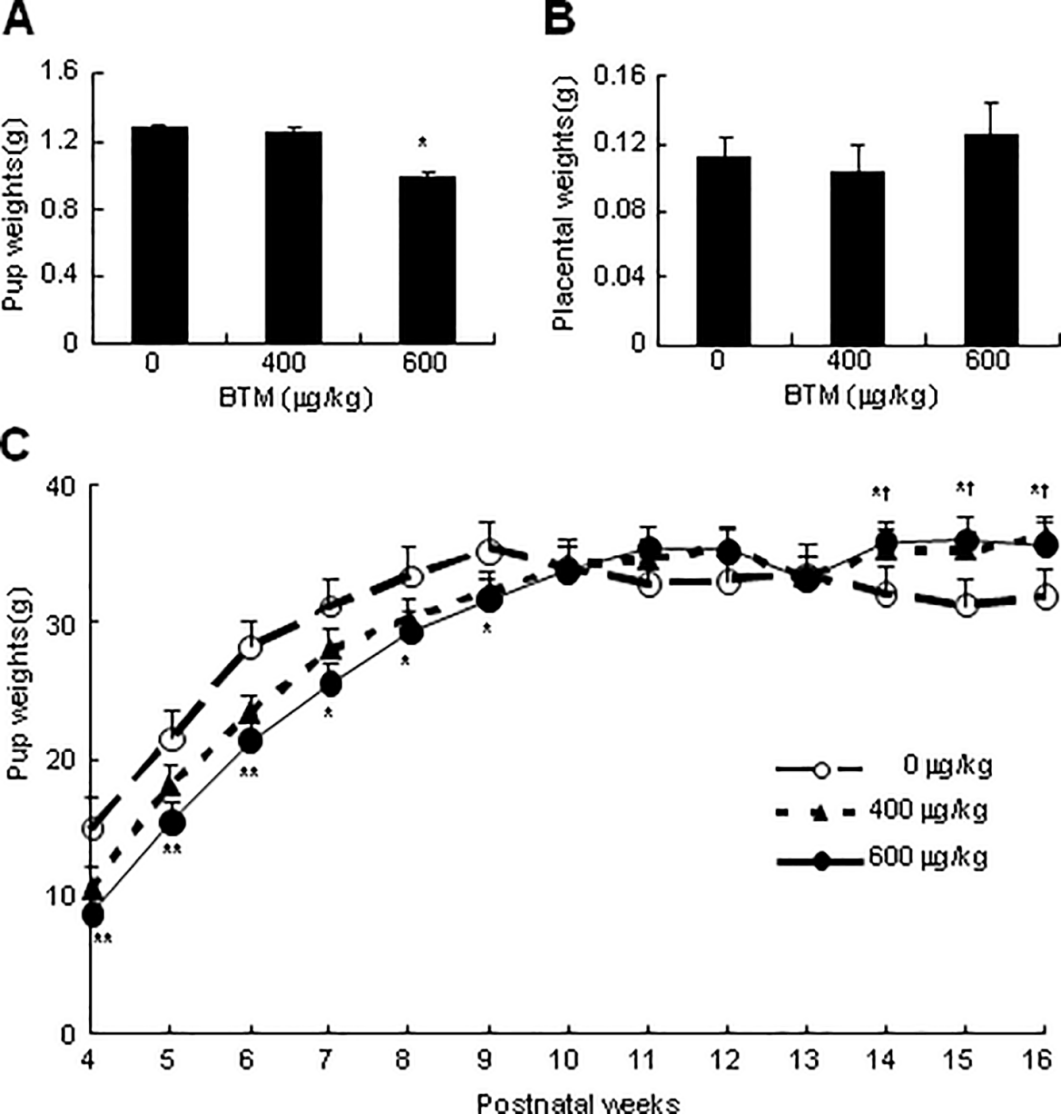
Effects of exposure to BTM on weights of placentas and offspring. Pregnant dams (each n = 12) were subcutaneously injected with BTM at 0, 400 or 600 μg/kg/d for 4 d from E14.5 to E17.5, and the fetuses (**A**) and placentas (**B**) (45 samples for each treatment) from 6 littermates were harvested and weighed at E18.5, respectively. After weaned, the pups (42 samples for each treatment) from the rest 6 littermates were fed with high-fat diets, and were weighed in indicated stages (**C**). Data are expressed as means ± SEM. *p < 0.05, **p < 0.01, BTM at 600 μg/kg/d versus at 0 μg/kg/d; ^†^*p* < 0.05, BTM at 400 μg/kg/d versus at 0 μg/kg/d.

To examine the long-term effects of exposure to BMT on the weights of placentas and offspring, we administrated the pregnant dams with either NS or BTM at 400 or 600 μg/kg/d for 4 d (from E14.5 to E17.5). The placentas and intrauterine fetuses were weighed at E18.5, and postnatal offspring were weighed in different developmental stages after weaning. The fetal weights were significantly declined at E18.5 in the pregnant dams challenged with BTM at 600 but not at 400 μg/kg/d (Fig 3A), and the placental weights in the pregnant dams treated with either 400 or 600 μg/kg/d of BMT were not significantly changed (Fig 3B). Exposure to BMT at 600 but not 400 μg/kg/d led to the significant reduction in body weights of the postnatal offspring younger than 10-week-old, whereas BMT at either 600 or 400 μg/kg/d led to the significant increases in body weights of the postnatal offspring older than 14-week-old (Fig 3C). Interestingly, a decline with no significance in the body weight in the control group after 14-week could be observed as well.

### Effects of exposure to BTM on glucose homeostasis and insulin secretion

**Fig 4:**
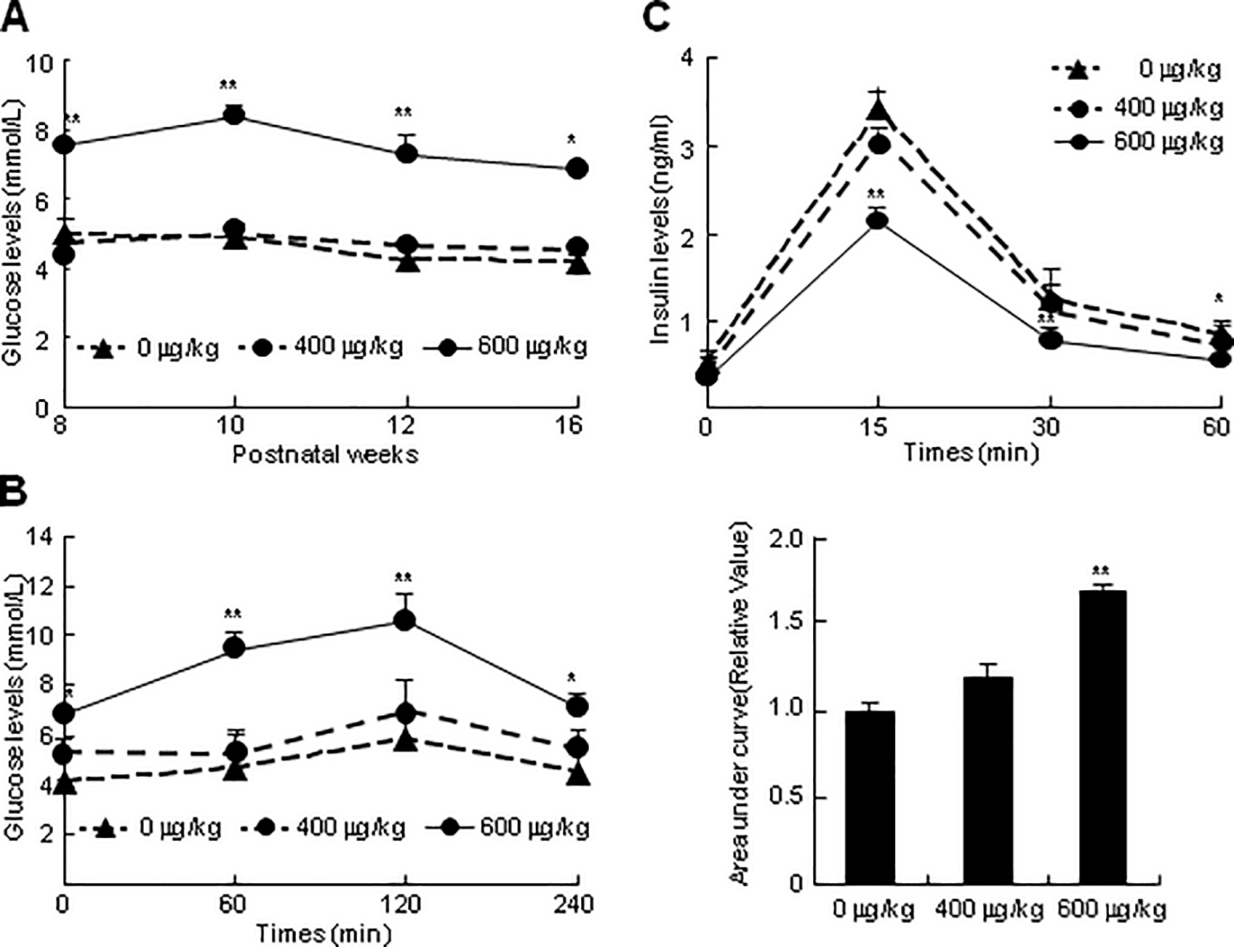
Effects of antenatal exposure to BTM on glucose homeostasis and insulin secretory capacity of the adult offspring. Pregnant dams were subcutaneously injected with BTM at 0, 400 or 600 μg/kg/d (each n = 12) for 4 d from E14.5 to E17.5. After weaned, the pups were fed with high-fat diets, and were subjected to further assays. (**A**) Serum glucose determination. The adult offspring (42 samples from 6 littermates for each treatment) at different ages were fasted for 12 h, and were subjected to isolation of sera for determination of glucose levels. (**B**) Glucose tolerence test and (**C**) insulin secretory capacity. Analyses of the area under the glucose tolerance test curve were provided (**B**, right). The adult offspring (42 samples from 6 littermates for each treatment) at 16-week-old were fasted for 12 h, and were orally administrated with glucose at 2 g/kg. 0, 1, 2 and 4 h after administration, the sera were isolated for determination of glucose levels; 0, 15, 30 and 60 min after administration, the sera were isolated for determination of insulin levels. *p < 0.05, **p < 0.01 versus antenatal exposure to BTM at 0 μg/kg/d.

To examine the effects of antenatal exposure to GC on glucose homeostasis and insulin secretory capacity, we determined serum glucose levels, glucose tolerance and insulin secretory capacity in adult offspring fasted for 12 h. Within 8 to 16 postnatal weeks, the fasting glucose levels were significantly higher in the offspring exposed to BTM at 600 μg/kg/d than in those exposed to BTM at 400 μg/kg/d or NS (Fig 4A). In the glucose tolerance test, within 4 h, the serum glucose levels in the 16-week-old offspring exposed to BTM at 600 μg/kg/d were significantly higher than those in the offspring exposed to BTM at 400 μg/kg/d or NS (Fig 4B). In the insulin secretory capacity tests, fasting insulin levels were quite low, but exhibited no significant difference in the offspring antenatally exposed to NS, BTM at 400 μg/kg/d or BTM at 600 μg/kg/d, however, glucose challenges led to significantly lower insulin secretion in the offspring exposed to BTM at 600 μg/kg/d than in those exposed to BTM at 400 μg/kg/d or NS (Fig 4C).

### Effects of exposure to BTM on serum corticosterone and lipid levels

**Fig 5:**
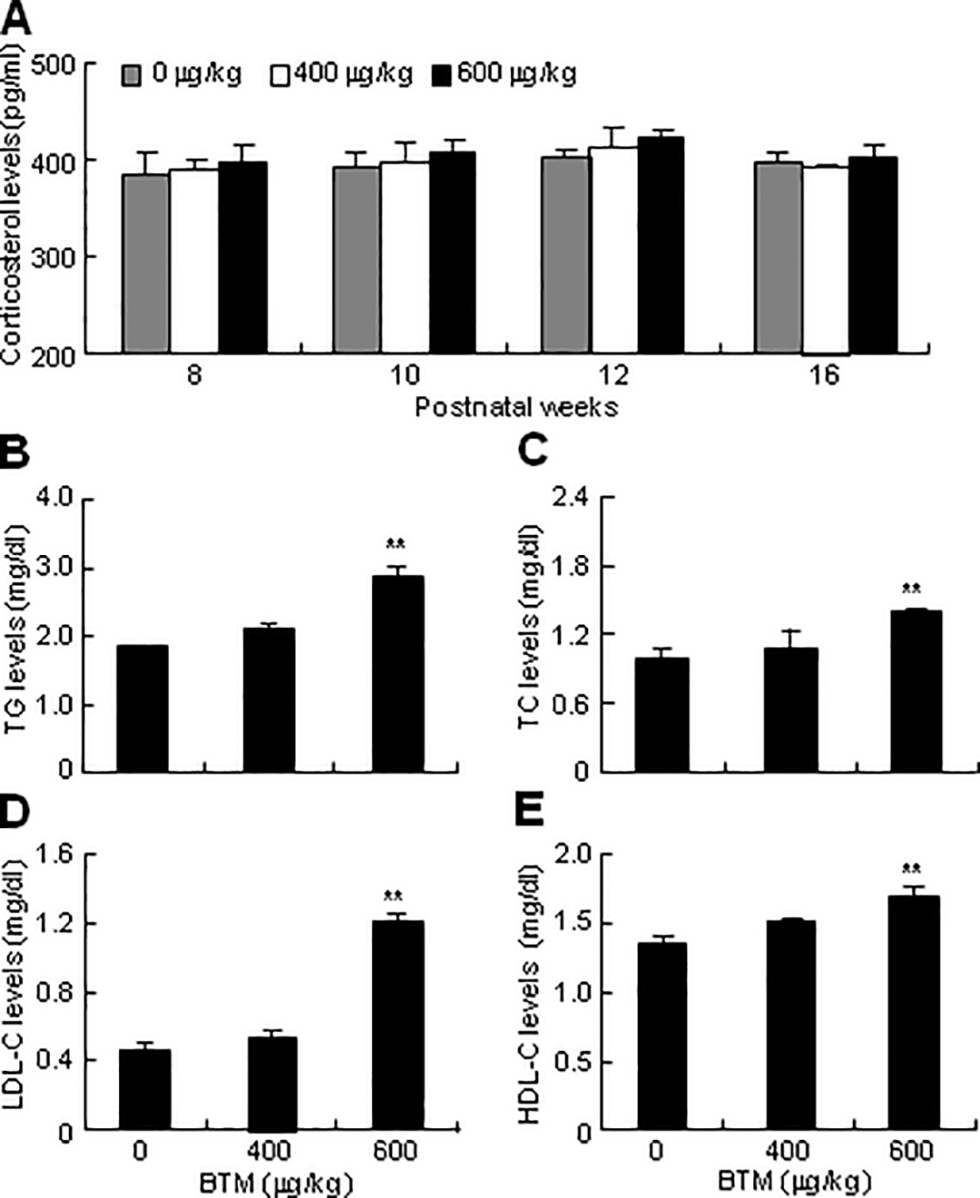
Effects of antenatal exposure to BTM on serum corticosterone and lipid levels of adult offspring. Pregnant dams were subcutaneously injected with BTM at 0, 400 or 600 μg/kg/d (each n = 12) for 4 d from E14.5 to E17.5. After weaned, the pups were fed with high-fat diets, and were subjected to further assays. At 16-week-old, the offspring (45 samples for each treatment) were sacrificed for determination of serum corticosterone (**A**),TG (**B**), TC (**C**), LDL-C (**D**) and HDL-C (**E**) levels. Data are expressed as means ± SEM. **p < 0.01 versus BTM at 0 μg/kg/d.

To evaluate the effects of antenatal exposure to BTM on corticosterone and lipid homeostasis in adult offspring, we performed serum corticosterone and lipid determination in the 16-week-old offspring exposed to BTM at 600 μg/kg/d, BTM at 400 μg/kg/d or NS. The corticosterone levels exhibited no significant difference between the offspring exposed to BTM at 600 μg/kg/d, BTM at 400 μg/kg/d and NS (Fig 5A). However, the serum TG, TC, LDL-C, and HDL-C levels were significantly higher in the offspring prenatally exposed to BTM at 600 μg/kg/d than in those prenatally exposed to BTM at 400 μg/kg/d or NS (Fig 5B–5E).

## Discussion

The present study examined the localization of 11β-HSD1 and 2 in murine placenta, and demonstrated that exposure of pregnant dams to BTM induced the expression of placental 11β-HSD2 but not 11β-HSD1, led to fetal IUGR and caused adult metabolic disorders. To our knowledge, we suggested, for the first time, that BTM induced 11β-HSD2 expression in murine placenta, and that antenatal exposure to BTM resulted in the disorders of glucose and lipid homeostasis in adult offspring.

11β-HSD1 is mainly distributed in the decidua and placental spongiotrophoblast layer in the early gestational stages, and is diffusely expressed in the murine placenta of late gestational stages. However, 11β-HSD2 expression is restricted to labyrinthine layer of murine placenta (Fig 1). These findings are consistent with previous literature that describes the expression patterns of 11β-HSD1 and 2 in placentas [25]. We suggest the expression patterns of 11β-HSD1 and 2 correspond well with their functions that 11β-HSD1 in decidua guarantees enough active GC to maintain local immunosuppressive effects for the blastocyst implantation and immunological tolerance to the alien tissue [3, 4, 26], and that 11β-HSD2 in labyrinthine layer inactivates the active form of GC to form a placental GC barrier protecting the embryo from the maternal GC attack during embryonic development [27]. However, the physiological significance of placental 11β-HSD1 expressed in placental spongiotrophoblast layer of early stages and in both spongiotrophoblast and labyrinthine layers of late stages remains unclear. We suppose that the expression patterns of 11β-HSD1 are consistent with the developmental patterns of murine placenta, and the expression of 11β-HSD1 could provide enough GC for the development of placenta itself. Further experiments need to be set up to test this hypothesis. In addition, the expression of placental 11β-HSD2 reaches the peak at E13.5, and then significantly declines with advancing gestation (Fig 1). This expression pattern of 11β-HSD2 corresponds with the developmental patterns of placental labyrinthine layer which originates from chorioallantoic attachment at E8.0, and is maturated at around E13.5 [28]. The expression levels of placental 11β-HSD2 in different gestational stages are also well consistent with the maternal serum GC levels which rise from the non-pregnant value of 2.3 μg/dl to 15.2 μg/dl on d 10, and further rise to and reach a peak of 138.3 μg/dl on d 16 [29].

Interestingly, antenatal exposure to BTM at 600 μg/kg/d increases 11β-HSD2 but not 11β-HSD1 expression at protein level (Fig 2). We suppose that the increase of 11β-HSD2 expression is resulted from the induction of its mRNA levels, since VE Murphy *et al*. proposed that BTM-induced 11β-HSD2 expression was caused by increased 11β-HSD2 transcription, enhanced mRNA stability and prolonged mRNA half-life mediated by activated glucocorticoid receptor (GR) [30]. Correspondingly, previous literature reported that maternal BTM administration dramatically increased 11β-HSD2 but not 11β-HSD1mRNA and protein levels in the baboon placentas [31]. However, another study described that significant increases in placental 11β-HSD2 mRNA expression was found in rats treated with dexamethasone, whereas this alteration was not observed at protein levels [32]. The inconsistency among different studies could be due to the difference in the animal strains, duration of GC administration, and dosing and timing of exposure during gestation. The pharmacological significance for BTM-induced 11β-HSD2 expression is of great interest, we suppose that BTM-induced 11β-HSD2 expression results in the increased of 11β-HSD2 activities to counteract the effects of GC, and to further protect the fetus from the maternal GC attack, which could be a self-defensive mechanism for the organism.

It has been well established that intrauterine exposure of GC resulted in IUGR [9], and this effect is observed in the present study as well (Fig 3), exhibiting that exposure of pregnant dams to BTM at term gestation significantly decreases the fetal but not the placental weights. Though the precise mechanism has not been clearly investigated, at least, exposure of fetus to excessive GC depressing fetal adrenal function was one important reason for GC-induced IUGR, as described previously [33]. In the present study, the lower body weights exist in the offspring antenatal exposure to BTM at 600 μg/kg/d but not 400 μg/kg/d, and last until 9-week-old, whereas antenatal exposure to BTM at either 600 μg/kg/d or 400 μg/kg/d leads to significantly higher body weights in more than 14-week-old offspring (Fig 3). In humans and rodents, the acute effects of GC manifest in weight loss rather than weight gain, whereas chronic GC excess in human, such as Cushing’s syndrome and metabolism syndrome, manifests in long-term weight gain [34, 35]. Thus, we suggest that the turnover of the body weights in the adult offspring between 9- and 14-week-old could signal the occurrence of metabolic syndrome. This hypothesis is consistent with clinical findings that weight change is a predictor of incidence of metabolic syndrome and remission of insulin resistance [36], and is further attested by our findings that in 16-week-old offspring, the weight gain is closely related to the glucose intolerance, low insulin secretory capacity and high levels of serum lipids (Figs 4 and 5), which are the properties of metabolic syndrome [37].

Antenatal exposure to BTM at 600 μg/kg/d led to hyperglycemia, glucose intolerance and low capacity of insulin secretion in the 16-week-old offspring (Fig 5). These findings are consistent with the mounting previous evidence that subtle abnormalities in metabolism occurred in the adult offspring prenatally overexposed to GC [38]. Many potential mechanisms contribute to GC-producing glucose intolerance in later life. In the rodents, prenatal exposure to BTM facilitates BTM passage to the fetus [39, 40], and BTM inhibits insulin release and islet beta-cell replication *in vitro*, thus, excessive exposure to GC may permanently reduce beta-cell mass, and result in impaired glucose tolerance [41]. In addition, GC opposes the effects of insulin leading to GC-induced insulin resistance which diminishes the suppression of glucose production and reduces peripheral glucose uptake [42]. Furthermore, the prenatal “programming” effect of GC is another potential mechanism underlying GC-induced disturbance of glucose homeostasis, and several definite candidate genes for programming effects, such as hepatic phosphoenolpyruvate carboxykinase (PEPCK) and 11β-HSD1 are regulated by GC through GR [43].

Up to date, antenatal overexposure to GC-induced disorders of lipid homeostasis in the adult offspring has not been well investigated, and to our knowledge, the present study, for the first time, shows that prenatal GC overexposure induces the adult offspring hyperlipidemia manifesting as high levels of serum TG, TC, LDL-C and HDL-C. These data partially correspond to the previous studies showing that GC administration in the patients results in a significant increase of serum HDL-C but not TC, TG and LDL-C levels [44, 45], the subtle difference between GC administration and prenatal GC exposure in disorders of lipid homeostasis is not well defined, however, we suppose that high levels of HDL-C but not TC, TG or LDL-C in GC-administrated patients could be due to the direct effects of GC on lipid metabolism, whereas high levels of serum TG, TC, LDL-C and HDL-C in animals antenatally exposed to GC could be resulted from “programming” effects of GC on the genes of lipogenase and lipase. Unlike Cushing’s syndrome that exhibits an increase in circulating very-low density lipoprotein (VLDL) and LDL, but not HDL, with consequent elevation of TG and TC levels [38], prenatal GC exposure-produced disturbance of lipid homeostasis in later life of offspring could not be resulted from the high serum corticosterone levels, since the present study shows that prenatal exposure to BTM at 600 mg/kg/d leads to no significant changes in serum corticosterone levels of 16-week-old offspring (Fig 5). It is plausible to suggest that overexposure of fetus to GC may permanently “programming” the abnormalities of lipid metabolism in adult offspring [43]. Previous studies indicate that GC induces several “programming” genes encoding enzymes in hepatic *de novo* lipogenesis and TG synthesis, including acetyl-CoA carboxylase 1 and 2, which encode rate-controlling enzymes in the fatty acid synthesis pathway, and fatty acid synthase (FASN), which encodes another rate-limiting enzyme in lipogenesis in many tissues [46–48]. However, the precise mechanism underlying the prenatal “programming” effects of GC has not yet been clearly investigated. Undoubtedly, there exists many key players involved in the postnatal metabolic syndrome, among which, the epigenetic mechanism, such as histone acetylation and DNA methylation, may help to shed light on many aspects responsible for the long-term “programming” of disease, and may provide great hope for therapeutic intervention [49]. In addition, given that GC has diverse effects other than up-regulation of placental 11β-HSD2, the observed IUGR and metabolic phenotypes in offspring could be interpreted with appreciation of other mechanisms besides placental 11β-HSD2.

## Conclusions

In conclusion, exposure of pregnant dams to BTM during the term gestation results in IUGR and induction of 11β-HSD2 but not 11β-HSD1 expression in placenta, and leads to the disorders of glucose and lipid homeostasis in adult offspring, providing evidence of 11β-HSD2 in fetal origins of adult diseases.
